# One fish, two fish, red fish, dead fish: Detecting the genomic footprint of ecological incompatibilities

**DOI:** 10.1371/journal.pbio.3001504

**Published:** 2022-01-11

**Authors:** Jenn Coughlan

**Affiliations:** 1 Biology Department, University of North Carolina, Chapel Hill, North Carolina, United States of America; 2 Department of Ecology and Evolutionary Biology, Yale University, New Haven, Connecticut, United States of America

## Abstract

As we uncover the ubiquity of hybridization in nature, determining how natural selection acts on hybrids has newfound importance for speciation. This Primer explores a study in PLOS Biology that uses threespine stickleback to detect a genomic signature of ecological incompatibilities.

## Teaser

As we uncover the ubiquity of hybridization in nature, determining how natural selection acts on hybrids has newfound importance for speciation. A study in *PLOS Biology* uses threespine stickleback to detect a genomic signature of ecological incompatibilities.

## Selection against hybrids takes many forms

A long-standing goal in evolutionary biology is to understand how natural selection acts on hybrids and assess how often selection against hybrids can contribute to reproductive isolation among species. Selection against hybrids is often classified as intrinsic or extrinsic based on whether hybrids exhibit low fitness across many environments or only in certain conditions, respectively [1]. These 2 mechanisms of selection against hybrids are also thought to have different genetic architectures. Intrinsic incompatibilities largely arise through structural rearrangements or multilocus incompatibilities, wherein novel allelic combinations in hybrids have an epistatic effect on fitness [1]. Extrinsic postzygotic isolation may stem from either an epistatic or additive genetic architecture. Most often, extrinsic barriers are discussed in terms of hybrid intermediacy, wherein hybrids exhibit intermediate phenotypes of locally important traits and are consequently maladapted to both parental environments (e.g., [2–4]). Yet, extrinsic barriers can also arise if multilocus genotypes give rise to transgressive traits or trait combinations that are deleterious in certain environments.

Although the idea that selection against hybrids may stem from these “ecological incompatibilities” is certainly not new [5], empirical examples are few and often involve F_1_ hybrids with mismatched traits. These mismatches may occur because which parent an F_1_ resembles can differ between ecologically important traits (e.g., dominance mismatches [6]). Dominance mismatches are likely common between ecologically divergent species ([6]; examples include [7,8]). However, if the genetic architecture of ecological divergence is largely additive, trait mismatches may not manifest until recombination and independent assortment have reshuffled alleles to create individuals with alternate homozygous ancestry at 2 or more loci (akin to a fully recessive intrinsic incompatibility).

While an intriguing possibility, the standard of evidence required to demonstrate such complex extrinsic incompatibilities is substantial; researchers must generate hundreds of F_2_ hybrids through manipulative crosses, rear replicate hybrid populations in multiple environments, then track traits and fitness for each individual in each environment to generate fitness landscapes that are environment specific.

**Fig 1:**
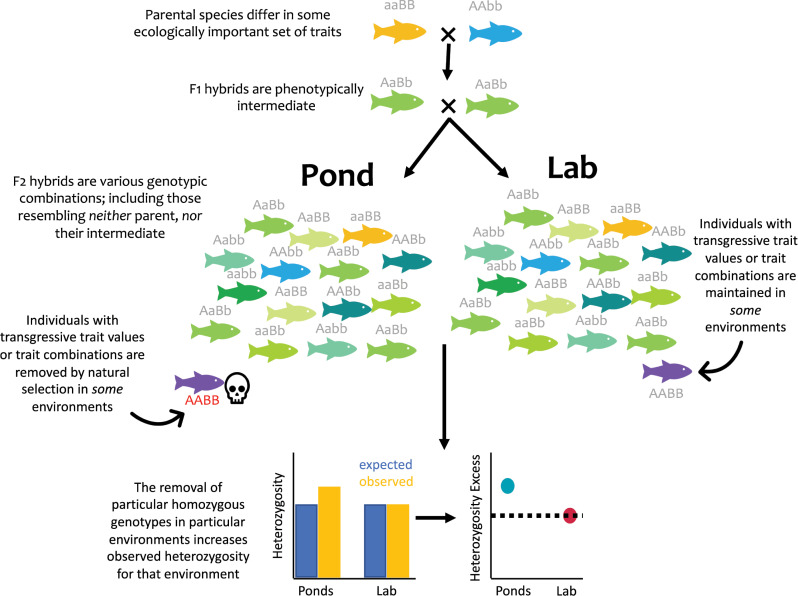
Ecological incompatibilities should leave a detectable genomic signature. F_2_ hybrids between 2 ecologically divergent species are raised in both benign lab and natural or seminatural field environments. If the genetic architecture of divergence is largely additive, then most hybrids will exhibit an intermediate set of phenotypes. Yet, a subset of individuals with largely alternate homozygous ancestry at different loci may exhibit trait mismatches or transgressive traits (shown in purple). If these individuals are selected against, then heterozygosity of the surviving hybrids will be elevated relative to the expectation based on allele frequencies. Under a model of ecological incompatibility, this excess heterozygosity should be observed only in natural environments.

Yet, selection against hybrids in an F_2_ generation is also predicted to leave distinct genomic signatures. If particular combinations of homozygous genotypes lead to lowered fitness (whether intrinsically or through ecological selection; Fig 1; [9,10]), then when those individuals are removed by natural selection, the surviving individuals will exhibit higher heterozygosity than expected at those particular loci. The more loci involved in the incompatibility, the stronger this genome-wide signature will be. This framework opens the door to a much simpler approach to detect ecological incompatibilities: Using ancestry-informative sites, compare heterozygosity of hybrids raised in the lab to those raised in natural conditions. If selection is ecological, then only hybrids raised in natural conditions should exhibit excess ancestry heterozygosity. The simplicity of this approach allows researchers to detect potential ecological incompatibilities in systems in which measuring multiple traits and tracking fitness per individual may be infeasible, or the specific traits involved are not obvious. Despite its promise, whether such signatures will be readily apparent at a coarse, genome-wide scale is largely unknown (though see [10]).

## Identifying the genomic signatures of ecological incompatibilities

In this issue of *PLOS Biology*, Thompson and colleagues build on previous efforts in threespine stickleback (*Gasterosteus aculeatus*) by creatively leveraging previously published studies and unpublished data to look for genomic signatures of ecological incompatibilities [11]. Previous work provided strong evidence that ecological selection against hybrids between limnetic and benthic forms of threespine stickleback may be caused by trait mismatches [12]. In this system, the genetic architecture of divergence is complex, involving many small-effect and largely additive loci. Intriguingly, a subset of F_2_ hybrids exhibit unusual jaw morphologies that combine distinct traits from either parental species (rather than intermediate phenotypes, which most hybrids exhibit; [12]). These trait mismatches do not impede feeding in the lab, but when raised in experimental ponds, these fish exhibit low feeding performance and consequently small body size [12].

To identify genome-wide signatures of ecological incompatibilities, Thompson and colleagues calculated how much heterozygosity pond-raised and lab-raised hybrids exhibited, and how much these levels of heterozygosity deviated from expected (i.e., excess ancestry heterozygosity [11]). Under a model of ecological incompatibilities, only the pond-raised hybrids should exhibit excess ancestry heterozygosity ([10]; Fig 1). In line with this hypothesis, the authors found that pond-raised hybrids were consistently more heterozygous at ancestry-informative sites, while lab-reared hybrids showed no deviations from expectation. Their results are robust; holding up across sequencing methods, studies, ponds, and even the parental populations used to create the hybrids. Moreover, because field-specific heterozygosity excess can be generated by several processes, the authors do a commendable job at ruling out alternative explanations, including field-specific heterosis and inbreeding depression. This work represents a significant step forward for speciation genetics, presenting evidence for ecological incompatibilities in a model system and also providing a straightforward method for detecting these types of incompatibilities in other systems.

## What, when, why, and how: Moving toward mechanism and assessing commonality

With new tools in hand, what are the next steps for understanding ecological incompatibilities? While Thompson and colleagues present a straightforward method for detecting ecological incompatibilities, amassing more examples will require substantial effort, and in many systems will be unachievable. Nonetheless, as more examples of ecological incompatibilities accumulate, 2 key areas of research will emerge: What are the mechanisms underlying ecological incompatibilities and how important are they for speciation?

Determining how selection acts on specific traits/trait combinations will be essential to understand how selection is acting against hybrids. This will require the nitty-gritty of building environment-specific fitness landscapes and tracking both the targets and agents of natural selection in hybrid populations. Moreover, a comprehension of the genetic architecture of the trait(s) involved in ecological incompatibilities will be a key step in elucidating the importance of this reproductive barrier. Understanding if the traits involved in ecological incompatibilities have largely additive genetic architectures with epistatic effects on fitness, the number and effect size of the loci involved, and the strength of epistatic selection can give insight into how much of the genome is expected to be involved in reproductive isolation, as well as how many hybrid individuals—and in what generation—low fitness is expected to manifest. The answers to these questions have crucial implications for the efficacy of ecological incompatibilities to limiting gene flow in nature. Lastly, assessing how common these types of incompatibilities are and when in the speciation process they tend to evolve are central for determining their importance in speciation. The latter 2 goals will require amassing many examples of ecological incompatibilities, both across taxonomic groups as well as lineage pairs of differing divergence times within a group. While much work is needed, the creative reuse of previously published datasets presented by Thompson and colleagues is a reminder that applying genomic technologies to previous experiments may serve as a fruitful avenue forward.
